# Is the Alma Ata vision of comprehensive primary health care viable? Findings from an international project

**DOI:** 10.3402/gha.v7.24997

**Published:** 2014-08-21

**Authors:** Ronald Labonté, David Sanders, Corinne Packer, Nikki Schaay

**Affiliations:** 1 Faculty of Medicine, Institute of Population Health, University of Ottawa, Ottawa, Ontario, Canada; 2 School of Public Health, University of the Western Cape, Cape Town, South Africa

**Keywords:** primary health care, health for all, community health workers

## Abstract

**Background:**

The 4-year (2007–2011) *Revitalizing Health for All* international research program (http://www.globalhealthequity.ca/projects/proj_revitalizing/index.shtml) supported 20 research teams located in 15 low- and middle-income countries to explore the strengths and weaknesses of comprehensive primary health care (CPHC) initiatives at their local or national levels. Teams were organized in a triad comprised of a senior researcher, a new researcher, and a ‘research user’ from government, health services, or other organizations with the authority or capacity to apply the research findings. Multiple regional and global team capacity-enhancement meetings were organized to refine methods and to discuss and assess cross-case findings.

**Objective:**

Most research projects used mixed methods, incorporating analyses of qualitative data (interviews and focus groups), secondary data, and key policy and program documents. Some incorporated historical case study analyses, and a few undertook new surveys. The synthesis of findings in this report was derived through qualitative analysis of final project reports undertaken by three different reviewers.

**Results:**

Evidence of comprehensiveness (defined in this research program as efforts to improve equity in access, community empowerment and participation, social and environmental health determinants, and intersectoral action) was found in many of the cases.

**Conclusions:**

Despite the important contextual differences amongst the different country studies, the similarity of many of their findings, often generated using mixed methods, attests to certain transferable health systems characteristics to create and sustain CPHC practices. These include:

1.  Well-trained and supported community health workers (CHWs) able to work effectively with marginalized communities
2.  Effective mechanisms for community participation, both informal (through participation in projects and programs, and meaningful consultation) and formal (though program management structures)
3.  Co-partnership models in program and policy development (in which financial and knowledge supports from governments or institutions are provided to communities, which retain decision-making powers in program design and implementation)
4.  Support for community advocacy and engagement in health and social systems decision making

These characteristics, in turn, require a political context that supports state responsibilities for redistributive health and social protection measures.

Primary health care (PHC), as the key health system strategy for attaining optimal health, gained global prominence with the 1978 Alma Ata Declaration (1). Its strategic role was reaffirmed 30 years later in the 2008 World Health Organization (WHO) World Health Report (2), with added weight given to its importance by the publication in that same year of a PHC theme issue by *The Lancet*(3). PHC has since received considerable attention in light of a final push toward the 2015 Millennium Development Goals (notably, the maternal and child health goals) and recent emphasis on achieving universal health coverage (UHC) (4), which is likely to be prominent in the post-2015 development goals.

Although the PHC strategy codified at Alma Ata was comprehensive – emphasizing the integration of rehabilitative, therapeutic, preventive, and promotive interventions, and attention to local social and environmental risks – it is generally acknowledged that this approach failed to be adopted as widely as anticipated (2, 5). Several reasons for this have been proffered, including a rapid return to selective PHC as a ‘temporary’ measure due to the costs of a more integrated strategy (6), the 1980s debt crises and rise of structural adjustment policies emphasizing public sector restraint and market-driven reforms (7), and the 1990s and 2000s surge in disease-specific global health initiatives that reinforced vertical interventions rather than horizontal health systems strengthening (8).

Different countries or groups throughout the developing world, however, continued over this period to strive toward the goals of Alma Ata, several examples of which were noted in the 2008 World Health Report (2). In 2005, prior to development of this Report, the Peoples’ Health Movement (PHM) in collaboration with other partners, being aware through their global networks of continuing efforts to influence comprehensive PHC, began a project to document these initiatives. With funding from the Canadian Global Health Research Initiative,^1^ we undertook an ambitious 4-year research program (2007–2011) to explore these initiatives in low- and middle-income countries (LMICs) in a novel structured design involving teams of junior researchers, research mentors, and research users (policy makers or service managers). This design simultaneously enhanced the capacities of young researchers while securing the participation of officials who are able to act upon the findings of the studies. The aims of the studies that comprised this research program were thus threefold: strengthening PHC research capacities in LMICs, documenting and assessing progress toward comprehensive PHC, and building linkages between researchers and health systems decision makers.

## The research program

A core group of seven senior researchers from Canada, Australia, Africa, and India met in late 2005 to design the program. Based on the original Alma Ata Declaration and their knowledge of PHC across all five continents (including developments in health promotion and human rights post Alma Ata), they developed a set of output and outcome criteria against which the comprehensiveness of PHC could be assessed, namely:

1. increased equity in access to health care and other services or resources essential to health
2. reduced vulnerabilities through increases in community empowerment (community capacities)
3. reduced exposures to risk through changes in social and environmental determinants of health
4. improved participatory mechanisms and opportunities, and increased political capabilities, of marginalized population groups reached by PHC initiatives
5. increased intersectoral policy actions on the social and economic determinants of health that involve the health sector
6. improved population health outcomes and greater health equity.

The first five of these criteria were hypothesized to be associated with the final outcome related to equitable improvements in health status. The research program would assess this association through a structured narrative literature review undertaken in the first year (for reports on these findings, see Refs. (9–11)), and it would focus new studies primarily on the first five PHC outputs.

The core team drafted a call for new research projects that would include research users as well as researchers in all phases of the study, and would examine PHC projects (or components of projects) that fulfilled two or more of criteria 1–5 of this list. These criteria were considered by the core team to represent an ideal level of comprehensiveness. There was no expectation that these criteria were likely to be fully realized in any one country or project, but that there would be evidence of efforts to include several of them in PHC programs.

The call for new research projects was widely distributed in English and Spanish through the PHM listserv. We received 85 Expressions of Interest (EOIs), only some of which came from members who were active in the PHM and others from teams that had learned of our research program through networks of the funding organizations and of the two program co-leads (Labonté and Sanders). Following a transparent peer review process, five projects were selected in each of the four global regions (Africa, Asia, Latin America, and Indigenous communities in Canada, Australia, and Aotearoa/New Zealand).^2^ Intensive 2-week meetings were held in each of the four regions with the selected projects, in which training was provided on research methodology, methods, ethics, and theories of health systems policy and community empowerment, as well as the development of detailed research plans. In each subsequent year, a weeklong meeting was reconvened in each of the regions to assess progress and develop analytical and knowledge dissemination strategies. The intensity of these annual capacity-enhancing sessions ensured to the furthest extent possible that findings were based on rigorous design and analyses. Each team had a senior researcher, and the final proposals for each project were subject to intense reviews by the overall research program co-leads, the senior research mentors, and all participating junior researchers and research users. Although the diversity and experience of the research triads varied across the sites, variations in rigor were minimized. Research activities for each of the projects spanned between 24 and 30 months. A final weeklong meeting was held in 2011 involving all 20 research teams, during which identification and initial analyses of cross-cutting themes took place.

A university research ethics board or relevant research association approved each detailed research plan of the different projects.

## The research projects

Despite their breadth, there was agreement among all of our research triads that our five outputs accurately captured what they believed to be important elements of comprehensive PHC. As Table 1 shows, projects approached their examination of this comprehensiveness differently. These differences reflected how the research triads responding to our EOIs identified what knowledge was needed and of specific interest to them regarding efforts to advance comprehensive PHC within the localities, regions, or countries in which their studies took place. Several studies focused on community health workers (CHWs), which is unsurprising given the importance of CHWs in comprehensive PHC. Other projects examined broader aspects of health systems approaches to comprehensive PHC, using historical or comparative case study methods or quantitative observational designs.

**Table 1:** CPHC revitalizing health for all research projects

Country (and City or Region, if Applicable) and Project Title	Research Team	Research Focus and Project Publications	Key Methods	CPHC Foci*
India (Bihar) The Contribution of Accredited Social Health Activist (ASHA) under National Rural Health Mission (NRHM) in the Implementation of Comprehensive Primary Health Care in East Champaran District, Bihar (State), India	Vandana Kanth, Jameela George, and Anil Cherian	The role of community health workers (ASHAs) in improving access to health services and advocacy on social determinants of health	Survey of 299 households to measure knowledge, behavior, and practice changes attributable to ASHA interventions Structured key informant interviews of ASHAs (199), child development workers (255), auxiliary nurse midwives (17), and village headmen and village committee members (27) Focus groups with village members	1, 2, 3, 4, 5
India (Arunachal Pradesh) Exploring Synergies between Empowerment and Gender and Comprehensive Primary Health Care in Three Tribal Districts of Arunachal Pradesh	Tage Kanno, Manjunath Shankar, Betsy Taylor, and Hage Tam	The role of village health volunteers in promoting gender empowerment and community participation, and its impacts on the comprehensiveness of and access to PHC services	Key health indicators (control and gender empowerment intervention localities) Key informant interviews (*n*=47) Participant observation (5 sites, multiple days) Focus group discussions (4 focus groups, total *n*=60) Photovoice project and analysis (3 sites)	1, 2, 3, 4, 6
Iran The Contribution of Community Health Workers to the Implementation of Comprehensive Primary Health Care in Rural Settings	Sara Javanparast, Gholamreza Heidari, and Fran Baum	The role of *behvarz* (Farsi term for community health workers) in enhancing all outputs of comprehensive PHC (1–3)	Systematic literature review Document analysis, including Iranian health ministry policy and unpublished reports Semistructured interviews with 91 *behvarz* in a representative sample of 18 Iranian provinces	1, 2, 3, 4, 5
Pakistan Perceptions about the Effectiveness of Three Models of Comprehensive Primary Health Care in Urban Squatter Settlements of Karachi	Parvez Nayani, Agha Ajmal, and Yousuf Memon	Comparison of the comprehensive and sustainability of three models of PHC service development in poor communities, supported by Agha Khan University: institution led (externally financed, planned, and delivered), community led (joint institution and community financed, community planned), and co-partnership (externally financed, community planned and managed)	Document analysis Key informant interviews (*n*=30) Focus group discussions (12 focus groups, total *n*=115)	1, 2, 3, 4, 5
Bangladesh Revitalizing Health for All: Developing a Comprehensive Primary Health Care Model for Bangladesh	Taufique Joarder, Aftab Uddin, and Anwar Islam	Identifying structural characteristics of comprehensive PHC through comparison of high- and low-performing health districts	Document analysis and 4 key informant interviews (for the history of PHC in Bangladesh) Index ranking of 10 urban and 10 rural health districts by health system performance using World Health Organization criteria from its 2000 report (for selection of high- and low-performing health districts) Ethnographic observations at two selected health districts, key informant interviews (*n*=40), randomized household survey (*n*=225 households) evaluating PHC services, and participatory rapid appraisal (*n*=30 participants) in each health district	1, 2, 3, 4, 5, 6
Uruguay Rural and Community Policlinics in Uruguay: Their Role in the New Integrated National Health System	Fernando Borgia, Ingrid Gabrielzyk, Jorge Soto, Marcela Azambuja, Alexis Gularte, Helena Giménez, Miguel Corneo, Marlene Arrarás, and Sebastián González	The contribution and role of rural and community policlinics (health centers) in the country's new National System for Integrated Health	Microdata from four waves of household survey (2007–2009) Systematic document review and analyses Administrative data and historical narratives from 119 policlinics representing urban and rural settings Data from five regional policlinic meetings and from 11 local forums in 19 states (*n*=over 900 participants)	1, 3, 4, 5
El Salvador The Experience of Primary Health Care in El Salvador	Areglia Dubón Abrego, Dagoberto Menjívar López, Eduardo Espinoza Fiallos, and Christa Baatz	A detailed historical case study of the comprehensive PHC system developed in the village of Guarjila (1987–2007), now serving as a model of PHC development nationally	In-depth interviews with community leaders and organized groups; and focus groups with community members Documentary and bibliographic analysis Participant observation	1, 2, 3, 4, 5, 6
Colombia Learning from the Experience of Primary Health Care in Bogota	Roman Vega, Paola Mosquera, Jinneth Hernández, C. Junca, and Jorge Martínez	The contribution of recent comprehensive PHC reforms in reducing health inequities through ecological analyses comparing high and low coverage in two disadvantaged localities, and pre- and post- implementation of PHC reforms, on child health outcomes (4, 5)	Data from two household surveys (2003 and 2007) and public health administrative databases calculating concentration curves and indices, and regression analyses, related to child health outcomes and selected indicators of PHC coverage Semistructured key informant interviews (*n*=18) and 14 focus groups (*n*=7–10/group) in district health localities, including decision makers, health staff, and community members	1, 2, 4, 6
Brazil Health Care Coordination: Construction of Comprehensive PHC in Brazil	Patty Fidelis de Almeida, Lígia Giovanella, and Berardo Augusto Nunan	Comparison of coordination mechanisms in four municipalities to integrate Brazil's CPHC model (Family Health Teams) into secondary and tertiary levels of health care (6, 7)	Health system administrative database Key informant interviews with all level of health staff, including community health workers (*n*=77)	1, 4, 6
Argentina The Political Dimension of Primary Health Care (PHC) in Argentina: Repressed Components, Disordered Growth and Models in Conflict: A Reconstruction from the Testimonies, Historical Footprints and Clues in a Temporal-Spatial Exploration	Mario Rovere, Andrea Jait, Analía Bertolotto, Ana Fuks, and Eugenia Bagnasco	Historical comparative study of different models of PHC programs targeting disadvantaged urban and rural populations (8)	Bibliographic review and analyses Key informant interviews (*n*=35) Focus group discussions (3 focus groups, total *n*=82) Discourse analysis	1, 2, 4, 5
Ethiopia (Jimma) Health Services Extension Program and Comprehensive PHC	Mirkuzie Woldie, Morankar Sudhakar, and Abera Assefa	The role of community health workers and community participation in improving health outputs and outcomes	Representative household survey in 3 districts at early, mid-, and late implementation of program (*n*=695) Key informant interviews (*n*=4) Focus group discussions (5 focus groups, total *n*=43)	1, 2, 4, 5
Ethiopia (Tigray) The Contribution of the Health Extension Program in Promoting Comprehensive Primary Health Care in Tigray, Ethiopia: The Case of Maternal Health	Araya Abrha, Mark Spigt, Yohannes Tewelde, and Yemane Berhane	The contribution of the Health Extension Program to the comprehensiveness of PHC services targeted to maternal health (9)	Representative survey of women in areas served by program (*n*=725) Key informant interviews (*n*=15) Focus group discussions (8 focus groups, total *n*=73)	1, 2, 4, 5
South Africa The Gauteng Province Community Health Worker Programme: The Extent to Which It Contributes to the Provision of Comprehensive Primary Health Care	Nonhlanhla Nxumalo, Jane Goudge, Liz Thomas, and Salamina Hiahane	A comparison of enablers and barriers to the effectiveness of community health workers in facilitating community participation, intersectoral action on social determinants of health, and access to formal health systems in three disadvantaged regions	Key informant interviews (*n*=24), including 4 focus group discussions Ethnographic participant and observation (74 household and CHW observations) Network mapping	1, 4, 5
Kenya Uptake and Contribution of a Community Strategy in the Renewal of Comprehensive Primary Health Care (CPHC) in Different Socio-demographic Contexts in Kenya	Jack Buong, Clementine Gwoswar, and Dan Kaseje	An assessment of the contribution of the Community Health Strategy (a model of CPHC now being generalized across the country) to the comprehensiveness of health system services in three sociodemographic contexts, and under three different modes of implementation	Household survey (*n*=3,694) Key informant interviews (*n*=7) Focus group discussions (14 focus groups, total *n*=134) Client exit interviews (*n*=422)	1, 2, 3, 4, 5
Democratic Republic of Congo Exploring the Complex Contributions of a Community Based Safe Motherhood Program to Comprehensive Primary Health Care	Jean Robert Likofata Esanga, Gwendolyn J. Lusi, and Richard Bitwe	Evaluation of a maternal health program (Safe Motherhood) based upon local organizations (solidarity groups, comprising microcredit, income generation, and pooled-insurance groups) for its contribution to maternal health outputs, and its potential as a model for health systems reconstruction in a postconflict setting (10)	Survey of solidarity group (*n*=174) and matched controls (*n*=174) Key informant interviews (*n*=6) 20 focus group discussions (solidarity group and control group members, traditional birth attendants, and men in both solidarity group and control communities)	1, 2, 3, 4
Australia (Alice Springs) Ingkintja: Learning from Comprehensive Primary Health Care Experiences	Clive Rosewarne, Gai Wilson, and John Liddle	Evaluation of the comprehensiveness of an Aboriginal-controlled male health program in a central Australian town (11)	Document and program evaluation analyses Key informant interviews (*n*=20) Community consultations (3 separate consultations, total *n*=39) Service user consultation (1 consultation, total *n*=12)	1, 2, 3, 4, 5
Australia (Utopia) Wellbeing at Utopia: The Role of Urapuntja Heath Service	Sarah Doherty, Kevin Rowley, and Ricky Tilmouth	Historical study of the comprehensiveness of an Aboriginal remote health service, serving the medical and social health needs of 700 Aboriginal people spread over 250 km^2^ (11)	Administrative data Census data (cross-sectional analysis) Archival document analyses Key informant interviews (*n*=11) Focus group discussions (2 focus groups, total *n*=16) Participant or observation	1, 2, 3
Australia (Melbourne) Victorian Aboriginal Health Service: Revitalizing Health for All Project	Bronwyn Fredericks, Joanne Luke, and Alan Brown	Historical study of the comprehensiveness of a community-run Aboriginal health service in an urban setting (11)	Administrative data Archival document analyses Key informant interviews and oral histories Focus group discussion	1, 2, 3, 4, 5
Aotearoa/New Zealand Setting Up the Try! The Role of the Community Health Worker in a Māori Person's Health Journey	Tania Forrest, Pat Neuwelt, Rowena Gotty, and Sue Crengle	The role of community health workers in improving access for Māori to culturally appropriate primary health care services	Administrative data Archival document analyses Key informant interviews (*n*=5) Focus group discussions (2 focus groups, total *n*=10)	1, 2, 4
Canada Comprehensive Primary Health Care in the Island Lake Communities: What Does It Mean and How Does It Look?	Marcia Anderson DeCoteau, Grace McDougall, Carly Scramstad, and Alex McDougall	Identification of Aboriginal health beliefs and values to inform a governance model for Aboriginal-run Comprehensive PHC services (12)	Literature review Focus group discussions (3 focus groups, total *n*=23)	1, 3, 4

CPHC, comprehensive primary health care; PHC, primary health care.

* Numbers refer to the output and outcome criteria of comprehensive primary health care used to guide the overall study.

1 Javanparast S, Baum F, Labonté R, Sanders, D. Community health workers’ perspectives on their contribution to rural health and well-being in Iran. Am J Public Health 2011; pp. e1–e6.

2 Javanparast S, Baum F, Labonté R, Sanders D, Heidari G, Rezaie S. A policy review of the support for and activities and outcomes of the community health worker program in Iran. J Public Health Policy 2011; 7: 1–14. DOI: 10.1057/jphp.

3 3. Javanparast S, Baum F, Labonté R, Sanders D, Rajabi Z, Heidari G. The experience of community health workers training in Iran: a qualitative study. BMC Health Serv Res 2012; 12: 291. DOI: 10.1186/1472-6963-12-291.

4 Mosquera P, Hernández J, Vega R, Martínez J, Labonté R, Sanders D, et al. The impact of primary healthcare in reducing inequalities in child health outcomes, Bogota – Colombia: an ecological analysis. Int J Equity Health 2012; 11: 66.

5 Mosquera P, Hernández J, Vega R, Martínez J, Labonté R, Sanders D, et al. Primary health care contribution to improve health outcomes in Bogota-Colombia: a longitudinal ecological analysis. BMC Fam Prac 2012. DOI: 10.1186/1471-2296-13-84.

6 Almeida PF, Fausto MCR, Giovanella L. Fortalecimento da atencao primaria a saude: estrategia para potencializar a coordenacao dos cuidados. Rev Panam Salud Publica 2011; 29: 84–95.

7 Almeida PF, Giovanella L, Mendonca M, Escorel S. Challenges for healthcare coordination: strategies for integrating levels of care in large cities. Cad Saude Publica 2010; 26: 286–98.

8 Rovere M, Laub C. Possibles: Una Publicacion Para Pensar Lo Local Junto A Lo Global. Buenos Aires: Associación Civil El Agora; 2009.

9 Medhanyie A, Spigt M, Kifle Y, Schaay N, Sanders D, Blanco R, et al. The role of health extension workers in improving utilization of maternal health services in rural areas in Ethiopia: a cross-sectional study. BMC Health Serv Res 2012; 12: 1–9.

10 Mihanda RW, Likofata JR, Luis GJ. Contribution of safe motherhood solidarity groups in using and accessing maternity services during a period of armed conflict. Health 2013; 5: 1085–91.

11 Fredericks B, Legge D. Revitalizing health for all: International Indigenous Representative Group learning from the experience of comprehensive primary health care in aboriginal Australia – a commentary on three project reports. Lowitja Institute; 2012. Available from: http://www.lowitja.org.au/sites/default/files/docs/Revitalising_Health_report.pdf

12 Scramstad C. Comprehensive primary health care in the Island Lake communities: what does it mean and how does it look? Department of Community Health Science, University of Manitoba; 2012.

Most of the project studies generated findings related to the five outputs (and, in some cases, health outcomes) that defined our research program.

## Methods

Table 1 provides detailed information on the methods used by each of the different projects. In general, most projects used mixed methods, including secondary data analysis, policy document reviews, key informant interviews, focus groups, and, in some instances, new surveys. The synthesis of findings presented in this article, and the details presented in Table 1, were created by a systematic review of final project reports undertaken separately by two of the authors (Labonté and Packer) and a student research assistant, and then compared to reach consensus.

## Results and discussion

1. Increased equity in access to health care and other services and resources essential to health

Our research program considered this output as the baseline for comprehensive PHC: necessary, but insufficient in the absence of actions specifically related to the other outputs below.^3^ Findings generally noted that the PHC services they were studying succeeded in improving access. Although few of the projects specifically analyzed the equity dimension (e.g. did access disproportionately increase for those in greatest need?), all of the PHC services being studied were targeted at areas or population groups experiencing both need and relative disadvantage:

- Immunization and health checkups were brought to villages by health extension workers (HEWs), removing the need for long walks to health outposts; and volunteer female CHWs increased family planning usage and HIV testing (Jimma, Ethiopia).
- Accredited social health activists (ASHAs) are increasing awareness of health services and improving access to facilities for pregnant women (Bihar, India).
- Groups of women, supported by seed funding to create solidarity groups, developed insurance funds to assist pregnant women to access maternity facilities. Utilization of maternity services by solidarity group members was 98%, compared to 27% of non–solidarity group members living in the same area (Democratic Republic of the Congo (DR Congo)).
- Communities more fully implementing a community health strategy showed greater immunization coverage, antenatal care, use of insecticide-treated nets (ITNs), vitamin A uptake, and proportion of households with safe water access (Kenya).
- Giving Aboriginal men ‘someplace to hang out’ in a male-only drop-in facility increased their access to, and increased utilization 13-fold of, primary care services, and it increased enrollment in violence intervention and prevention counseling programs (Alice Springs, Australia).
- Cultural sensitivity training for comprehensive PHC teams increased service use for migrant and indigenous populations and improved mutual trust between service providers and community members (Argentina).

In Iran, the *behvarz* program (consisting of male and female trained and paid CHWs operating from ‘health houses’) was found to significantly increase coverage of health care throughout rural areas. The project documented how this coverage gradually expanded beyond infectious diseases and maternal and child health to include elderly care, mental health, chronic disease management, oral health, and youth health.

Although most projects showed improved access, they also documented shortcomings. For example, failure initially to align more closely with the work of traditional birth attendants (TBAs), who remain the primary resource for care in childbirths in many low-income countries, slowed the uptake of maternal and child health programs in Jimma, Ethiopia. The economics of user fees also came up as an issue: health center delivery in DR Congo costs US$5.00, which is unaffordable to many women, whereas TBAs delivering in the village are paid with a chicken. The poor physical quality of health centers dissuades women from giving birth in these centers (in Ethiopia and DR Congo). Childbirth and postnatal care still occur primarily within the home with TBAs, rather than in a health post attended by an HEW, a finding that is attributed to the lack of privacy or comfort in the posts and little confidence in the better trained though inexperienced HEWs (e.g. in Tigray, Ethiopia).

Safety concerns with travel to health centers was also a barrier noted in several of our projects, such as military conflict in DR Congo, or the continuation of armed conflicts in some parts of Ethiopia. Safety was also an issue in some cases for health workers, such as the female CHWs in one of the rural Bangladesh districts in the delta region, where travel by boat was risky and drowning accidents were frequent.

Although most projects noted improvements in the geographic accessibility of health care, others found a majority of households still far from health centers. For instance, over 60% in the non-intervention sites in the Kenya study were more than an hour away, a figure that improved only slightly to 50% following the community health strategy intervention. While access to health facilities improved in both Bangladesh case study districts, individuals in the ‘better-performing’ district (which is close to the capital, and where populations are generally wealthier) had much shorter travel times to the facilities, indicative of continuing geographic disparities. Both districts are understaffed, although much more so in the ‘poorer-performing’ district. Nationally, the PHC reforms in Bangladesh still reach only a minority of the population. Central planning in the country can also create inequitable outcomes: as one example, each district (regardless of geography or population density) is allocated one ambulance, but the ‘poorer-performing’ district in the delta region has no roads for its ambulance and no assigned driver, and it would have benefitted much more by being allocated a water taxi. Often, access entitlements are inadequately provided, the result of chronic underfunding or a failure of the government to enforce its own regulations (e.g. in Uruguay). In Uruguay's case, this led to an increase in the use of privately insured services.

2. Reduced vulnerabilities through increases in community empowerment (community capacities)

There is a large health promotion literature on community empowerment and capacity building that distills to improving the knowledge, analytical skills, local leadership, internal and external resource mobilization, and organizing abilities of disadvantaged communities (12–15). The evidence-informed argument this literature presents is that improvement in these capacity (or empowerment) domains reduces vulnerabilities to disease threats by increasing both material and psychosocial resources for health. Most projects manifested some efforts in this direction, notably the gendered dimensions of empowerment:

- Women community health volunteers (supported by HEWs) create space for village women to learn about and share in confidence their reproductive health problems (Jimma, Ethiopia).
- Women's solidarity groups work with traditional male leaders to mobilize support for better health care access, and to challenge disempowering traditions (DR Congo).
- Residents in the ‘better-performing’ health district reported frequent health meetings, improved health knowledge, and high levels of satisfaction with the community meetings run by the facilities (Bangladesh).
- Providing a safe cultural space for Aboriginal men was important to counter the dominant negative stereotype of such men being violent, abusive, and substance abusing (‘men's self-esteem is so low … because they have been forgotten’), and allowed these men to explore the roots of these negative behaviors (Alice Springs, Australia).
- Maori CHWs assist Maori community members in developing critical health literacy, and challenging prejudice or racism they may experience in seeking health care to which they are entitled (Aotearoa/New Zealand).

The men's program in central Australia was notable for recognizing the importance of providing a safe cultural space for Aboriginal men, as well as for Aboriginal women, as was the DR Congo program for working with men to challenge traditional and disempowering patriarchal norms that threatened maternal health. The Colombia study found that communities receiving a model (comprehensive) PHC program, compared to the PHC program delivered through government or private facilities, demonstrated a much greater community orientation in all aspects of planning, coordination, and delivery, embodying some facets of empowerment.

Many of the projects simultaneously noted strengths (or possibilities) and weaknesses in PHC contributions to empowerment. Although many of the PHC services emphasized peoples’ rights and entitlements (e.g. the projects in South Africa, India, Bangladesh, and Kenya), this was compromised by low literacy rates (Bangladesh) or lack of knowledge among CHWs of their role in promoting such rights (Bihar, India). As another example, despite emphasis on gender empowerment, gender dynamics were sometimes more sidelined than dealt with explicitly. Husbands of members of the women's solidarity groups in DR Congo still expressed concern over their wives giving birth in a health center, partly because of the possibility of a male health worker seeing their wives naked. The gender empowerment project in Arunachal Pradesh, India, noted a concern that is well known in the community development literature: functioning women's groups took around 5 years of support to become sustaining, but rarely were resources available for this period of time.

3. Reduced exposures to risk through changes in social and environmental determinants of health

Although policy discourse on social determinants of health (SDH) has gained momentum following the work of the WHO Commission on SDH (16), the importance of actions on SDH has existed in the health promotion literature since the 1980s. It is also referenced in the 1978 Alma Ata Declaration itself, although not in the same terms and with an emphasis on improved sanitation and potable water, and on community development more generally. Several instances of modest interventions on SDH were documented by the research projects, some reflecting more traditional environmental actions and others incorporating local economic development initiatives:

- Villages with HEWs had seven fold greater rates of latrine versus open field use than the rural average for the country (Jimma, Ethiopia).
- HEWs facilitated income-generating activities (e.g. microfinancing, beekeeping, and poultry raising) (Tigray, Ethiopia).
- Over half of ASHAs are recruited from families living below the poverty line, and the payments they receive for specific interventions improve their own living conditions (Bihar, India).
- Women's empowerment groups were effective in improving social support and assistance in health care and local food security (Arunachal Pradesh, India).
- Household poultry raising and crop diversification (i.e. food security and income generation) increased with the introduction of a community health strategy, together with training programs on small-scale agribusiness (Kenya).
- Aboriginal people living in parks (homeless) are supported to find employment and housing (Melbourne, Australia).
- Advocacy by a health center–led community coalition to reduce alcohol availability led to a reduction in alcohol consumption, alcohol-related hospital admissions, and the number and severity of alcohol-related assaults (Alice Springs, Australia).

All of the projects in low-income countries included efforts to improve sanitary conditions and potable water access. Food security and food safety initiatives exist in some projects, such as in Iran, but they suffer neglect in many others, such as in Bangladesh, although food security and safety initiatives are strengthening in Bangladesh with efforts to create specially trained nutrition workers. The presumption of the Bangladesh nutrition program that education is the best means to ensure food security is moot, however, given the growing marginalization of small farmers in an increasingly globalized system of food production, trade, marketing, and consumption.

Sometimes, the improvements were subtle, as in the Safe Motherhood program in DR Congo where the open membership of the solidarity groups was regarded as effective in breaking down some of the social class stigmas that persisted in the region. In India, over half of all of the ASHAs who serve the poorest communities, and who receive some remuneration for doing so, themselves come from the poorest and most stigmatized socioeconomic group in the country. Comprehensive PHC, in its structure and staffing, has enormous potential to challenge structural inequities: witness the innovative use of, first, women health workers on bicycles, and then as drivers, and then as security guards and physicians (each new role challenging patriarchal norms) by the Gonoshasthaya Kendra (GK) NGO in Bangladesh.^4^ As issues of sanitation and water access ceased to dominate, housing and other SDH (e.g. exposure to unhealthy products) gained more prominence. When CHWs are paid (even if only for specific interventions delivered) and are recruited from the poorest population groups (as in Bihar, India), their own health and that of their family may improve.

One of the weaknesses cutting across all projects, however, with the exception of the community-managed and well-established Aboriginal projects in central Australia (Alice Springs) and Melbourne, was the localized nature of SDH initiatives. This is not unique to low-income countries, as our structured narrative literature reviews found, and it attests to the difficulties that local communities often experience in gaining access to the higher-level economic and policy decisions that condition and constrain their local community efforts.

4. Improved participatory mechanisms and opportunities, and increased political capabilities, of marginalized population groups

Few aspects of PHC have received as much study or commentary as community participation. As with CHWs, participation by community members is regarded as an axiomatic feature of PHC services. Participation, however, can take many forms from cooptation to complete control, as Arnstein's famous essay on citizen participation noted (17). Early on, in the efforts to ‘roll out’ comprehensive PHC, warnings were made about the use of volunteers to fill in for irresponsibly underfunded public health programs, or about the emphasis on community members as free labor in facility building or service delivery but not in decision making (18). For this reason, our initiative emphasized participation as a ‘political capability’, with some, but not all, projects showing movement in this direction:

- Village health committees organized community dialogue days, facilitated by volunteer and paid CHWs, to identify health concerns that are brought to health facilities and the subdistrict management levels, with an obligation to report back to the village committees regarding what actions would be taken. The project team attributed these features to the higher rates of PHC access and outputs experienced by villages implementing the full PHC community strategy, compared to those that did not (Kenya).
- Of three models examined in the Pakistan study, community-led and co-partnership comprehensive PHC models, but not an institution-led model, involved community members in management decision making. The community-led model had more active volunteers in health center activities, but the co-partnership model was the only one that mentored members on ‘making approaches to higher authorities’ and exercising their political rights (Pakistan).
- Community workers in the model PHC program are mandated to work with communities to identify their needs and to increase demand for services, as an entitlement (Colombia).
- All health facilities must display a Citizens’ Charter noting the health service and other entitlement rights that people have; notably, this was absent in the ‘low-performing’ health district in Bangladesh.
- With community members, the Victoria Aboriginal Health Service (VAHS) advocates for social services, housing, and employment benefits to ensure that Aboriginal entitlement rights are respected; and VAHS has been a catalyst for Aboriginal communities elsewhere to develop their own community-controlled health services (Melbourne, Australia).

The finding from the Pakistan project is particularly interesting. In keeping with what might be considered a participation mythology (i.e. of the autonomously functioning and self-reliant community), the research team originally hypothesized that the more sustainable and comprehensive program would be the ‘bottom-up’, community-led model. Its finding that it was the co-partnership model, however, is consistent with arguments that PHC services, as public goods, should represent an equalizing partnership between state (or NGO) funders and community organizations (19).

Participation in PHC, however, remained problematic in many of the projects. In the South African project, there was little recognition of the role that community participation could play in improving health or health system access, although one site operating through an NGO with a stronger history of community accountability did perform better. In Ethiopia, community participation was sometimes restricted to provision of volunteer labor in building latrines or other public health goods; and, although such participation improved with the HEW program, it remained weak, which was partly attributed to concerns with safety (Jimma, Ethiopia).

Our Argentinian study documented how participatory approaches to PHC were undercut by 15 years of military dictatorship and the adoption of neoliberal economic policies. The impact of these historical processes persisted in the practices and habits of health workers employed in PHC services, embodying two different approaches: the ‘military-epidemiological’ model (vector control) and the ‘model missionary promoter’ (primary care for the poorest but with little interest in the conditions of poverty). Both models are inherently authoritarian, a finding that has positive implications for a different approach to training and support to new PHC workers in the wake of the democratic and socially progressive reforms now characterizing much of the region.

The main structural element for both empowerment and participation in PHC is presumed to be community health committees that, among other tasks, are intended to manage CHWs and their work. But community health committees were often nonexistent (e.g. Bihar, India) or nonfunctional, with little or no support or training regarding their purpose of supervising CHWs (South Africa). Participation in such structures can be dominated by individual interests or, in the absence of a community history of participation in political processes, heavily influenced by local leaders or partisan groups (Colombia). Despite the popularity of community health meetings in Bangladesh, which focused on more passive forms of education, community members in both health districts reported virtually no mechanisms for their participation in health planning or management to register concerns or complaints at their health facilities. They expressed a keen desire for such a structure, albeit cautioning that it should not be overwhelmed by ‘elites’, such as doctors and other government health workers.

5. Increased intersectoral policy actions on the social and economic determinants of health that involve the health sector

Although aligning it primarily with traditional PHC concerns (e.g. education, sanitation, and safe water), the Alma Ata Declaration recognized the importance of intersectoral action for health (IAH). IAH (and related terms, e.g. ‘healthy public policy’ and ‘health in all policies’) underscores the importance of health systems engaging with other sectors of governance to improve the conditions that shape health opportunities. Evidence of health system work in IAH was found in only some of the projects:

- Although constrained by a lack of IAH at higher levels of the health system, HEWs created good linkages with the education and agricultural sectors, and they quickly became identified as the ‘go-to’ people if any sector needed support or cooperation from others (Jimma, Ethiopia).
- *Behvarz* are involved with other local sectors in environmental, sanitary, and food safety programs, and they have established a council through which they attempt to bring intersectoral issues to higher governance levels (Iran).
- Community members created a multilevel governance system to address conditions that were negatively affecting their health: they constructed a potable water system, houses, and latrines; developed a food production system; and created an educational model that was popular with teachers and community health agents and that promoted health and rehabilitation (Guarjila, El Salvador).
- Working with justice and legal services, VAHS documented and intervened in cases of discrimination against Aboriginals by the police, leading to improved police–Aboriginal relations (Melbourne, Australia).

IAH is closely linked with community mobilization on SDH, and it frequently implies advocacy for high-level policy changes that affect local-level SDH. As such, it is one of the most politically sensitive components of comprehensive PHC. It is also one of the most difficult to evaluate given the diverse influences that shape policy decision making.

In most projects, there was little evidence of intersectoral activities, with ‘every sector doing its own thing,’ even where there was some effort being made to increase the level of IAH (Jimma, Ethiopia). Structures for local-level intersectoral action exist, but fragmentation within local government services and a general lack of support to community-based organizations rendered them ineffectual (South Africa). In the case of ASHAs in Bihar, India, their earnings are based on specific activities (e.g. immunizations and clinic visits), which becomes a disincentive for any work related to community mobilization or intersectoral actions. ASHAs would prefer a salary to pay-for-performance. Despite some evidence of IAH in Bangladesh, these actions related primarily to assistance from local education facilities (for health education) and police and law enforcement groups (to enforce security around the facilities), and they were weakened by a lack of any guidelines from more senior government levels on the obligations that sectors have for mutual cooperation. Exceptions again existed with the Aboriginal projects, where community control and ‘ownership’ were given particularly strong emphases. In the Canadian case, such control was not yet present but was strongly voiced by community members. Although financed through government transfers, the presence of elected Aboriginal-majority boards of management in the Australian projects was considered fundamental to their ability to act on social determinants, engage in political forms of advocacy for IAH, and provide services extending beyond the purely medical.

6. Improved population health outcomes and greater health equity

As noted in this article, few of the projects were able to capture health outcomes, as distinct from health system outputs. Some findings directly generated, or assessed, by our projects include:

- Rural infant mortality in Iran fell from a 10-fold greater rate (1973) to being equal to the rate in urban centers (2003) in direct relation to the introduction of the rural *behvarz* program, with similar improvements in most other health indicators (in Iran), although other factors were undoubtedly also at play.
- Compared to other rural and remote communities, the Utopia community enjoys significantly better health indices, which are attributed to its integration of services and its incorporation of traditional Aboriginal cultural approaches to healing (Utopia, Australia).
- Households in the ‘high-performing’ health district were more likely to report good or moderate health compared to those in the ‘low-performing’ health district, which also did much more poorly on elements of CPHC (Bangladesh).
- Compared to similar villages and the national averages, key health indicators (infant and under-5 mortality, maternal mortality, prenatal care, and undernutrition) were substantially better, which was attributed to the comprehensiveness of the PHC interventions (Guarjila, El Salvador).
- Health indicators improved as the comprehensiveness of PHC services increased, with health inequalities (infant and under-5 mortality) also decreasing (Colombia).
- Some of the evidence was more anecdotal. As one long-serving VAHS health worker recalled, ‘it was common to see [Aboriginal] kids with running ears and noses in the area; you don't see that anymore’, a change that has been attributed to the presence of the Aboriginal-run community PHC program (Melbourne, Australia).

Two topics that merit separate consideration are the role of CHWs and PHC financing.

### The role of CHWs

We commented earlier on the emphasis that almost all of our projects gave to the role of CHWs. But although people trusted their health workers, there were also concerns with the inadequate level of their training. In the South African study, training was considered adequate, but the relationship of CHWs to the formal health system remained unclear. Their lack of ‘empowerment’ was seen as a crucial reason for their inability to engage with the communities they were intended to serve. But the study also found a keen interest to engage more in SDH work – with additional training and health system support. A similar finding emerged from the study of ASHAs in Bihar, India: most reported being inadequately trained in their roles but, when made aware of their intended responsibilities in community mobilization and social health work, expressed keen interest to undertake these activities, albeit on the assumption that they would receive appropriate training, remuneration, and village and health system support.

Although appropriate training remained a minor concern with the *behvarz* in Iran, where initial training was 2 years in duration with annual workshops, the larger issue was over their expanding roles, including responsibilities for community engagement, intersectoral work, and actions on SDH. A similar caution was noted by the HEWs in the Ethiopian studies, where their broadly defined role made them the ‘go-to’ people for any other government or sectoral activity requiring community engagement. Although such a role is consistent with the premise of comprehensive PHC, it nonetheless raises issues of adequate resourcing and supervision, and the status that CHWs are accorded within the formal health system. Although their status varied considerably across our project sites, even in Brazil, where CHWs are formally integrated in the family health teams, they often lack credibility and recognition, impeding their efforts to coordinate across health care levels. A basic question that was identified, but unresolved, by our research program is: are CHWs primarily the health system's representative in the community, or the community's activist representative to the health system? It was clear that there is a spectrum, ranging from an organic link with communities and multifaceted community development activities established over years in political struggle (as in El Salvador), to more of a ‘health service’ relationship (as in Brazil).

### The role of financing

In some of the low-income countries in our study, the need for international financing was always present. In Ethiopia, the HEW and CHV program depends upon international funding, often tied to HIV or vertical maternal and child health financing, and thus it is vulnerable to any reductions in health development assistance. In El Salvador, where the community had been excluded from government support for political reasons, international solidarity groups supported its activities financially. Even in DR Congo, and despite its promotion of market gardens and other means to develop income for the solidarity group community insurance, its Safe Motherhood program still relies on donations raised by the NGO (HEAL Africa) that initiated it.

Funding does not always have to be international, although reliance on local (community) financing can prove unsustainable, if not also inequitable, due to lack of financial cross-subsidization. The Pakistan study of three different models of CPHC in poor communities, for example, found that an externally financed and planned (top-down) CPHC model showed fewer signs of community participation or knowledge of and action on SDH than did a jointly financed and community-managed (bottom-up) CPHC model. But a co-partnership model, in which all of the financing was externally provided with the community still retaining planning and management of the services, showed most evidence of comprehensiveness, presumably because it did not have to focus its own limited resources on health care access only. In Bangladesh, publicly employed physicians were often absent from their post and servicing their private practices instead, partly a result of low-salary schemes that allowed public health doctors an amount of private practice time to supplement their incomes, and partly the outcome of little monitoring of provider services by health administrators. Other financing challenges encountered include rationalization of public funding, which in the case of Utopia could lead to services being centralized to larger rural centers and undermining the Aboriginal governance and design of Utopia's presently decentralized program.

Finally, as our DR Congo study found (and this was consistent across all of our studies), the concept of community self-sufficiency or self-reliance (where localities are seen as wholly responsible for all facets of their economic, social, and health provisioning) needs to be discarded and replaced by one that emphasizes community self-determination (where localities are capable of negotiating for the financial and technical resources required for such provisioning with governmental and nongovernmental actors). The external financial support received by the DR Congo project, however, was always channeled through formal community structures that allowed for local-level self-determination and authority. This finding is compatible with the increased attention now being paid to the need for international financing for health systems that extend beyond the traditional development assistance envelope. This attention is partly in recognition of the huge inequities in countries’ abilities to mobilize social protection revenues, the historical and ongoing role of international economic institutions in sustaining these inequities, and the moral and human rights obligations to rectify these preventable inequalities (20).

### Policy outcomes

One of the reasons for including research users as part of the triad, and emphasizing the importance of triads that have strong linkages to civil society, was to ensure that findings were quickly ‘translated’ (transferred or exchanged) into policy decision making. We cannot claim that our research program alone was the basis for the policy actions that arose, because some of these initiatives were already underway and were merely augmented by our research projects. Nonetheless, a number of developments promptly followed the studies supported by our project, examples of which include:

1. Increased and improved training for ASHAs (Bihar, India)
2. Prioritization of rural health and a National Rural Health Program as part of health systems reform (Uruguay)
3. Strengthening of policies concerning the integration of different levels of care within the Family Health Program (Brazil)
4. Adoption of Guarjila as the exemplar model for national PHC reforms (El Salvador)
5. Expansion and deepening of the model (comprehensive) PHC program (Colombia)
6. Greater attention by the provincial health authorities to community representative structures in PHC (Gauteng, South Africa)

Other policy effects are also anticipated from other projects, although at present we have no documentation of such outcomes.

### Limitations

Several projects used mixed methods, including new surveys or secondary data analysis, but few attempted to examine quantitatively many of the perceptions captured in the qualitative components (key informant interviews and focus groups) of their studies.

### Reflections on the program

Our project teams conducted regular self-assessments of the effectiveness of their partnerships in order to advance their projects’ potential to transform research into practice. This process began with a structured exercise during the second round of regional training sessions that focused on some of the key elements of the ‘knowledge translation and exchange’ (KTE) process. During the final regional workshops, teams were asked to reflect on their triad experience. The feedback from the teams on the triad experience was overwhelmingly positive. Most groups suggested that having individuals with different expertise enhanced the research relevance and usefulness as well as the ability to develop relationships and disseminate the research findings. The major negative factor lay in difficulties in communicating between triad group members due to differences in location and scheduling conflicts.

The involvement of the research-user was perceived as significantly increasing the opportunities and effectiveness of the program and for better relationships with both policy developers and local health workers. Many of the teams commented that the triad was a model that other researchers, government organizations, NGOs, and communities could use to strengthen their research-to-action, and to bridge effectively the worlds of people engaged in policy and program design and implementation, and those involved in generating research and evaluating evidence. One interesting and unexpected finding from the overall experience of the triads was that the distinction between researcher and research-user was often artificial and, in some cases, resented. ‘Research-users’ were often competent researchers themselves and not passive consumers of research knowledge or subordinate to the methodological expertise of researchers. Researchers, in turn, often had important insights into program and policy work. This in no way weakens the conceptual importance of establishing research triads, but it cautions that care and sensitivity are required when assigning the different labels. The inclusion of regional workshops within the research program (with some attracting several hundred participants) increased attention to the principles and practices of comprehensive PHC and the dissemination of findings from all projects as they were being generated. Finally, all of our research projects were short term and operated with very limited budgets; our entire program, including literature reviews, regional and global training programs, associated travel, and 20 new research projects, was financed with US$1.9 million. This meant that several of the studies were only examining in some depth only two or three aspects of a PHC program, albeit always with efforts to make some sense of the whole through local literature reviews and contextual analyses. Although we had anticipated a renewal of this research program to support teams and regions, the impact of the 2008 global financial crisis led to reductions in funding opportunities for such a continuation of a globally coordinated set of studies.

## Conclusions

As global discourse shifts from issues more directly related to health services and disease prevention to financing and ‘universal health coverage’ (UHC), it becomes more important to convey what is known about comprehensive PHC, and the political and social contexts that make it possible to thrive. A recent article on this topic argued that considerable confusion and controversy around PHC remain, including disagreements over whether PHC should:

1. emphasize selective (vertical) interventions with defined packages and outputs, or function as a comprehensive (horizontal) approach to overall health systems development;
2. incorporate actions on socioenvironmental risk conditions (SDH), or remain focused on individual risk factors and biomedical (clinically defined) conditions; and
3. be seen as the point-of-first-contact for ill individuals with the formal health system in which integration across care levels is emphasized, or as the locus through which health systems engage with communities and other sectors on broadly defined health concerns, including SDH (21).

These disagreements are not new, and they may be less dichotomous than how they are often presented, but nonetheless they continue to exert substantial influence over health care access, short- and long-term health outcomes, and how equitably resources for health are distributed across population groups.

Our research program offers a cautiously hopeful signal that PHC reforms are indeed moving in the direction of horizontal comprehensiveness, even amid a plethora of globally driven, vertical disease initiatives; are acknowledging (and initiating some actions on) SDH; and are making efforts (albeit sometimes weak) to engage communities on broader issues of health equity and the social economic and environmental conditions that improve it. Moreover, it also affirms the importance (noted by respondents in most of our studies) of a horizontal approach to PHC that incorporated actions on SDH and broad forms of community participation. As part of their study remit, each of our research teams was asked to review critically the reports on PHC literature pertinent to their region, to fill in any gaps (drawing extensively from the gray literature), and to elaborate on the specific historic and contemporary contexts in which their findings of PHC reforms were situated. Their final reports contain detailed contextual analyses, across which a consistent theme, and one consonant with our structured literature reviews (see previously cited articles and the database at http://www.globalhealthequity.ca/data/index.php), stands out: the importance of political commitments to health equity and to health reform policies that clearly identify primary care, community participation, and intersectoral action as essential health system components. These overarching conclusions from the synthesis of our wide range of studies have been confirmed in two recent key and influential publications, the *Lancet* series on PHC (22) and the World Health Report of 2008 (2). It is this political commitment to the ethos of comprehensive PHC that remains most important to its revitalization today.

## Key messages

1. The comprehensiveness of primary health care (PHC) is more likely in countries that include political commitments to equity, a legal or constitutional right to health guaranteed by the state, and commitments to universally funded health and social programs.
2. In countries where government funding for public health systems remains low, nongovernmental organizations (NGOs) play a prominent role and are more likely to emphasize participation, integration with health promotion, empowerment, development, and a rights-based approach than government-led programs.
3. A vision of comprehensive PHC, first formulated at Alma Ata, remains a vital element of health systems activism in many LMICs. Leadership and courage within government and civil society, however, are needed to restrain the ongoing intrusion of selective approaches, whether through global health initiatives, donor pressures, or an uncritical embrace of private-public partnerships.

## Footnotes

1 The research program was made possible through funding provided by the Teasdale-Corti Global Health Research Partnership Programme, a collaborative health research programme developed by the four founding partners of the Canadian Global Health Research Initiative – Canadian Institutes of Health Research, International Development Research Centre, Health Canada, and Canadian International Development Agency – with input from the Canadian Health Services Research Foundation. We acknowledge the support given by the facilitators of the project, most notably the University of Ottawa and the University of the Western Cape. Additional funding was provided by CORDAID, the Alliance for Health Policy and Systems Research, the International Development Research Centre, and the Lowitja Institute (Australia). Some projects (e.g. the study in Colombia and the Australian Indigenous studies) received small amounts of funding in addition to that provided by the research program. All teams, however, received approximately the same amounts of funding for their studies, adjusted for the purchase power parity of the countries in which the studies took place.

2 Two additional projects participated in different phases of the study with some additional financial support (one in Nicaragua and another in Bangladesh), but they are not included in this article's discussion of findings. The peer review process for selecting the projects used a formal protocol consisting of a set of criteria consistent with the terms of the program identified in the EOIs and a ranking system adapted from that used by peer review committees of the Canadian Institutes for Health Research. Peer reviewers included the core team and senior researchers in each of the regions who were not involved in submitting an EOI or in conflict with a particular EOI proposal.

3 We note disagreement among some of our research mentors over whether provision of health care services was necessary to defining comprehensive PHC, citing public health and health promotion initiatives (e.g. Healthy City or Healthy Community programs) that satisfied all of our outputs and outcome criteria except for provision of health care services. Although we accept that this is the case, our interest in this research program, and following the PHC definition of the Alma Ata Declaration, was in how *health services* engaged in these outputs, hence the primacy of this first output.

4 A historical case study of GK, with an analysis of one of its new rural developments, was undertaken alongside the international program, but it was not fully integrated within it. The case study is available at http://www.globalhealthequity.ca/webfm_send/287.
